# Ketogenic diet improves fertility in patients with polycystic ovary syndrome: a brief report

**DOI:** 10.3389/fnut.2024.1395977

**Published:** 2024-09-12

**Authors:** Yumiko Tsushima, Noura Nachawi, Kevin M. Pantalone, Marcio L. Griebeler, Ula Abed Alwahab

**Affiliations:** ^1^Department of Medicine, Diabetes and Metabolic Care Center, University Hospitals Cleveland Medical Center, Case Western Reserve University School of Medicine, Cleveland, OH, United States; ^2^Department of Metabolism, Endocrinology and Diabetes, University of Michigan, Ann Arbor, MI, United States; ^3^Endocrinology and Metabolism Institute, Cleveland Clinic Foundation, Cleveland, OH, United States

**Keywords:** ketogenic diet, fertility, polycystic ovary syndrome, obesity, ovulation, pregnancy

## Abstract

**Introduction:**

Polycystic ovary syndrome (PCOS) affects up to 20 % of reproductive-age individuals and is strongly linked to obesity. The impacts of ketogenic diet on fertility in people with PCOS are unknown. This study aims to determine the effect of a ketogenic diet on restoration of regular menstrual cycles and fertility.

**Methods:**

After approval from the Institutional Review Boards of Cleveland Clinic, a retrospective analysis was conducted using the electronic health record system. We analyzed data from thirty patients (n = 30) with polycystic ovary syndrome who followed a ketogenic diet for at least 3 months at the Cleveland Clinic, Cleveland, Ohio, USA. Main outcomes were percentage of women with restoration of regular menstrual cycles and pregnancy rate.

**Results:**

All women (*n* = 30) had restoration of regular menstrual cycles. The overall pregnancy rate of women desiring pregnancy (*n* = 18) was 55.6% (*n* = 10). Pregnancy rate was 38.5% for women on metformin and 100% for those who were not (*P* = 0.036). Pregnancy rate was 62.5% for women using ovulation induction agents and 50.0% for those who did not (*P* = 0.66). Percent weight change between the pregnant and non-pregnant groups did not significantly differ [−8.1 ± 6.2, vs −6.4 ± 8.4, *P* = 0.64, respectively].

**Conclusion:**

This study reports a higher rate of pregnancy with the ketogenic diet in women with PCOS compared to existing literature.

## Highlights:

• Obesity plays a key role in the mechanism of infertility affecting up to 60% of women with PCOS.
• Keto Diet (KD) improves characteristics of PCOS such as insulin levels, free testosterone, and LH/FSH ratio.
• The effect of Keto Diet on fertility in patients with PCOS is unknown.
• The KD may improve ovulation and fertility rates in women with PCOS regardless of metformin or ovulation induction use.

• Further prospective studies evaluating the impact of a KD on fertility appear warranted, as well as mechanistic studies to further elucidate the mechanism by which a KD may improve fertility, with or without weight loss.

## 1 Introduction

Polycystic ovary syndrome (PCOS) affects 6–20 % of reproductive-age women depending on the population studied and the diagnostic criteria used (1). It is one of the most common causes of anovulatory infertility in this population. PCOS is strongly associated with obesity and its prevalence in the PCOS population may be as high as 75% (2). Obesity, along with insulin resistance, plays a key role in the mechanism of infertility in these women, whereby an elevation in luteinizing hormone leads to increased circulating androgens which inhibits ovulation (3).

Diet-induced weight loss and insulin-sensitizing agents are shown to improve ovulation (2, 4). The ketogenic diet (KD) has garnered increasing attention for its ability to cause significant weight loss and improve metabolic syndrome, both of which are characteristics of PCOS. Currently, the term KD is used broadly because of its many variants. The original KD was designed to treat pediatric epilepsy and is composed of a 4:1 ratio of fat to protein plus carbohydrates (in grams) (5). Modified versions of this have been adapted for weight loss and diabetes. Feinman et al. have suggested that a very low carbohydrate ketogenic diet consists of 20–50 g of carbohydrates per day or carbohydrates < 10 % of a 2000 kcal per day (6).

Investigations regrading the effects of KD on patients with PCOs are limited to small and short-term studies. In a small study, patients with obesity and PCOs who underwent KD for 24 weeks, had significant reductions in body weight (−12%), fasting insulin (−54%), percent free testosterone (−22%), and LH/FSH ratio (−36%). Insulin, glucose, testosterone, HgbA1c, triglyceride, and perceived body hair didn’t differ from baseline. Two patients conceived despite previous infertility problems (7). Same changes were demonstrated in another study involving 12 patients with PCOs and overweight who underwent KD for 12 weeks had significant reductions in body weight, BMI, fat body mass and visceral adipose tissue with slight reduction in lean body mass. Significant reductions in insulin, glucose, HOMA-IR, total cholesterol, triglycerides, LDL, LH/FSH ratio, LH, total and free testosterone, and DHEAS levels were observed. HDL, estradiol, progesterone and SHBG levels increased. The study didn’t show any difference in Ferriman Gallwey Score (8).

However, the effect of a KD on fertility in patients with PCOS has not been previously reported. In a small case series, our group previously reported that the KD restored regular menses in all patients and achieved pregnancy in 50% of the cohort (9). The objective of this report is to build upon this previous report and provide additional evidence for the use of KD to restore regular menstruation and achieve pregnancies in women with PCOS.

## 2 Materials and methods

After approval from the Institutional Review Boards of Cleveland Clinic, a retrospective analysis was conducted using the enterprise-wide electronic health record system at Cleveland Clinic, Cleveland, Ohio.

Patients with PCOS who were referred to the Endocrinology and Metabolism Institute’s Integrated Weight Management Program for management of PCOS and obesity were identified. The Integrated Weight Management Program is comprised of monthly shared medical appointments (SMAs) of up to 10 patients who are evaluated using a multidisciplinary approach consisting of encounters with a registered dietitian who provides education and structured guidance on the KD plan, exercise physiologists for individualized exercise programs, and endocrinology and obesity specialists who lead the program.

The KD plan implemented in our SMA consists of up to 20 g of net carbohydrates, protein intake of 1.6 x weight in Kg = grams/day, Fat intake up to 40 grams/day. At least 64 oz of water intake is required. The use of ketogenic supplements was discouraged. However, potassium chloride supplement of 10 meq per day were prescribed. Calorie count was not required however, the above plan would reach 1000–1200 Kcal/day for all patients.

### 2.1 Study population

The electronic health record system at the Cleveland Clinic was queried for women who were enrolled in the SMA program for PCOS management from September 2017 to September 2019. The SMA program enrolls 50 to 60 patients per year and involves unlimited number of monthly visits. Of patients enrolled in the SMA program, women who were diagnosed with PCOS, had clear documentation of KD initiation, remained on the KD for at least 3 months, or were on a KD for less than 3 months but achieved pregnancy, were included. We excluded women who discontinued the KD prior to 3 months duration (for reasons other than achieving pregnancy), changed to a non-KD before 3 months or initiation date of the KD was not clearly documented, and those actively using contraception or were post-menopausal. Women receiving metformin and ovulation induction agents were also included. Thirty women who met the above outlined criteria were identified.

### 2.2 Intervention

Participants were enrolled in SMA program that included monthly visit. Exercise counseling was provided in the first visit without further monitoring. Participants had an initial visit with the physician and dietician at the start of the KD. Monthly visits were conducted to document changes in weight and menstruation and to address any dietary and medical concerns. Patients were given the option to start metformin if there was no prior history of intolerance. Ovulation induction agents were also offered to women seeking pregnancy. Patients were asked to notify the provider if they became pregnant or stopped the KD plan. Women who because pregnant were advised to switch to a healthy pregnancy diet plan provided to them on their initial visit with the dietician.

### 2.3 Outcome measures

The primary outcomes were (1) percentage of women with return to regular menstrual cycles and (2) pregnancy rates. Additional outcomes included time to return of regular menstrual cycles, time to conception, change in weight at return of regular menstrual cycles, and change in weight at conception. The primary outcomes were compared between women receiving metformin and not receiving metformin and between those receiving fertility induction and those who were not. Regularity of menstrual cycles was documented based on self-report of monthly cycles.

### 2.4 Statistical analysis

The study used a convenience sample of 30 women with PCOS that followed the KD for at least 3 months. A formal sample size calculation prior to starting the study was not performed. However, the chosen sample size of 30 allows for 80% power to detect moderate effect sizes of more than 0.5 standard deviations for continuous changes based on a paired *t*-test with 0.05 significance level. Categorical variables were described using frequencies and percentages, and comparisons between groups were assessed using Pearson’s chi square or Fisher’s exact tests. Continuous variables were described using means and standard deviations, or medians and quartile, based upon results of a Shapiro-Wilk test for normality and normality q-q plots. Comparisons between normally-distributed continuous variables were assessed using two sample *t*-tests with equal or unequal variance assumptions, as appropriate. Paired *t*-tests were performed to assess weight change within groups with regular period return and pregnancy. Kaplan- Meier estimates were calculated for time to return to regular periods and pregnancy, and Kaplan-Meier plots were used to show time to these events by Metformin and Fertility Induction use. Hazard ratios from Cox proportional hazards models were used to compare Metformin and fertility induction agent use on these outcomes. Analyses were performed SAS Software (version 9.4; Cary, NC).

## 3 Results

### 3.1 Patient characteristics

A total of 30 women met the inclusion criteria. Baseline patient characteristics are summarized in Table 1. The mean age of the cohort was 31.1 ± 4.9 years and mean BMI was 43.4 ± 9.1 kg/m^2^. Sixty percent of patients were receiving metformin. Sixty percent of the women reported desire for pregnancy.

**TABLE 1:** Patient characteristics.

	Total (*N* = 30)	
Factor	*N*	Statistics
**Patient Characteristics**		
Age*	30	31.1 ± 4.9
Race, *n* (%)	30	
Caucasian		24 (80.0)
African American		4 (13.3)
Other		2 (6.7)
Presence of acne, n (%)	29	12 (41.4)
Presence of hirsutism, n (%)	29	22 (75.9)
Testosterone (ng/dL)*	24	40.9 ± 28.3
Serum Anti-Mullerian Hormone (ng/mL)^ⱡ^	12	3.7 [2.5, 9.0]
Regularity of periods, n (%)	30	
Regular		6 (20.0)
Irregular		24 (80.0)
Desire for pregnancy, n (%)	30	18 (60.0)
Initial BMI*	30	43.4 ± 9.1
Metformin use, n (%)	30	18 (60.0)
HDL (mg/dL)*	13	45.8 ± 14.2
LDL 9mg/dL)*	13	116.1 ± 12.8
Triglyceride^ⱡ^	13	105.0 [80.0, 130.0]
**Induction Agent Use**		
None, *n* (%)	30	22 (73.3)
Clomiphene, *n* (%)	30	4 (13.3)
Letrazole, *n* (%)	30	5 (16.7)
FSH, *n* (%)	30	1 (3.3)
hCG, *n* (%)	30	0 (0.00)
Other, *n* (%)	30	0 (0.00)

*mean ± SD, ⱡ median [Q1, Q3].

### 3.2 Menstruation and weight

Approximately 92% of women who had irregular menstrual cycles had return of regular menstruation at 6 months and it was 100% at 15 months. Mean weight change was −7.12 ± 6.63 kg at return of regularity of menstrual cycles compared to initial weight (*P* < 0.001).

### 3.3 Pregnancy and weight

Eighteen of the 30 women desired pregnancy. In this sub-cohort, 10 women (55.6%) achieved pregnancy. Approximately 63% of those who successfully achieved pregnancy did so within 12 months. Women who became pregnant vs. those that did not, lost a similar amount of weight (−7.05 kg vs. −6.85 kg, respectively). The pregnant group achieved resumption of regular menstruation in an average of 81.4 ± 39.0 days compared to 143.6 ± 172.5 days in the non-pregnant group. The overall weight change is summarized in Table 2.

**TABLE 2:** Weight Change By Pregnancy Status.

Status	N	Initial Weight (kg)	Post Weight (kg)	Weight Change (kg)	Weight Change (%)	*P*-value
Pregnant	10	108.5 ± 22.9	99.4 ± 20.5	−9.1 ± 7.8	−8.1 ± 6.2	0.64^a1^
Not Pregnant	8	135.5 ± 29.0	126.4 ± 27.7	−9.1 ± 12.2	−6.4 ± 8.4	

Statistics presented as mean ± SD. *p*-values: a1 = *t*-test.

### 3.4 Effect of metformin

Eighteen out of the 30 women were receiving metformin (60%). All women who had irregular menstruation at the start of the KD achieved regularity regardless of metformin use. Mean weight change at return of regular menstruation was −7.8 ± 6.6 kg in the metformin group and −6.2 ± 6.9 kg in the no metformin group (*P* = 0.58). Mean time to return of regular menstruation was 71.5 [310, 88.0] days in the metformin group and 75.0 [47.0, 105.0] days in the no metformin group (*P* = 0.98).

In terms of pregnancy outcomes, 5 out of 13 (38.5%) patients in the metformin group achieved pregnancy and 5 out of 5 (100%) in the non-metformin group achieved pregnancy (*P* = 0.036). Mean change in weight at conception was −11.3 ± 9.6 kg in the metformin group and −6.9 ± 5.9 kg in the no metformin group (*P* = 0.40). Time to pregnancy was 223.0 [175.0, 232.0] days for the metformin group and 328.0 [223.0, 385.0] days in the no metformin group (*P* = 0.76) Table 3.

**TABLE 3:** Weight change by metformin use.

	Not on Metformin (*N* = 12)		On Metformin (*N* = 18)		
Factor	N	Statistics	N	Statistics	*P*-value
**Patient Characteristics**					
Initial BMI*	12	41.7 ± 10.8	18	44.6 ± 8.0	0.41^a1^
HbA1c*	9	5.7 ± 0.57	13	5.6 ± 0.33	0.49^a1^
**Return Of Regular Period**					
Return of regular menstruation, n (%)	10	10 (100.0)	18	14 (100.0)	N/A
Initial weight (kg)*	10	110.9 ± 33.7	14	122.3 ± 24.9	0.35^a1^
Weight at resumption of regular menstruation (kg)*	10	104.7 ± 29.9	14	114.5 ± 25.6	0.40^a1^
Initial weight to weight at return of regular menstruation *	10	−6.2 ± 6.9	14	−7.8 ± 6.6	0.58^a1^
Time to return of regular menstruation^ⱡ^	10	75.0 [47.0, 105.0]	14	71.5 [31.0, 88.0]	0.98^b^
**Pregnancy**					
Pregnancy, n (%)	5	5 (100.0)	13	5 (38.5)	*0.036* ^d^
Initial weight (kg)*	5	107.7 ± 26.2	5	109.3 ± 22.2	0.92^a1^
Weight at conception (kg)*	5	100.8 ± 21.7	5	98.0 ± 21.6	0.84^a1^
Initial weight to weight at conception*	5	−6.9 ± 5.9	5	−11.3 ± 9.6	0.40^a1^
Time to Pregnancy^ⱡ^	5	328.0 [223.0, 385.0]	5	223.0 [175.0, 232.0]	0.76^b^

*P*-values: a1 = *t*-test, b = Wilcoxon Rank Sum test, d = Fisher’s Exact test. *mean ± SD, ⱡ median [Q1, Q3].

### 3.5 Effect of ovulation induction agents

In the 18 women who desired pregnancy, 8 utilized ovulation induction agents. In the ovulation induction group, 5 out of 8 (62.5%) achieved pregnancy, and among those that did not utilize ovulation induction agents, 5 out of 10 (50%) achieved pregnancy (*P* = 0.66). Time to pregnancy was 223.0 [175.0, 232.0] days in the ovulation induction group and 385.0 [223.0, 453.0] days in the no ovulation induction group (*P* = 0.37). There was no statistically significant difference in pregnancy rates at 9 months whether metformin or ovulation induction agents were used.

### 3.6 Anti-mullerian hormone

Out of the 18 women who desired pregnancy, 10 had anti-mullerian hormone (AMH) values available for review. Out of the 4 women who became pregnant, only 1 woman used an ovulation induction agent. The lowest AMH value in the pregnant groups was 3.4 ng/mL and 0.1 ng/mL in the non-pregnant group. Two women with the lowest AMH values (0.1 and 2.1 ng/mL) were unable to become pregnant despite fertility induction Table 4.

**TABLE 4:** AMH by Pregnancy Outcome and Fertility Induction.

Patient	Fertility Induction	BMI	HbA1c (%)	AMH (ng/mL)
**Pregnant**				
1	N	40.8	6.6	3.4
2	N	46.8	5.2	3.6
3	Y	33.1	5.4	8.6
4	N	25.6	5.3	10.8
**Not Pregnant**				
5	Y	47.5	5.3	0.1
6	Y	54.0	6.3	2.1
7	N	40.8	5.1	5.1
8	N	65.6	5.7	9.4
9	N	25.6	5.3	10.8
10	N	40.8	5.3	23.7

N: no, Y: yes.

## 4 Discussion

This study examined the effect of a KD on menstruation and pregnancy rates in women with PCOS. All women who had irregular menstrual cycles achieved regularity and 58.8% of those who desired pregnancy became pregnant. The pregnancy rate of women who used ovulation induction agents (62.5%) was higher in our study compared to data from existing literature (10–12). The percentage of women who became pregnant without the use of ovulation induction agents (50.0%) was also higher than previously reported (10–12).

The prevalence of obesity in women with PCOS is high with some studies reporting up to 75% (2), and a pooled estimated prevalence of 49% according to a meta-analysis performed by Lim et al. (13). Obesity further worsens insulin resistance (14) which correlates with ovulatory dysfunction (15). In small studies, a KD was found to improve weight along with metabolic and endocrine parameters such as serum testosterone, serum insulin, and luteinizing hormone/follicle stimulating hormone ratio (7, 8). Physiology suggests that improvements in these parameters would also improve ovulation and fertility. However, studies evaluating the effect of weight loss on ovulation and pregnancy rates are lacking. To the best of our knowledge, our study is one of the first studies to address the effects of a KD on fertility in women with PCOS.

Legro et al. performed a post-hoc analysis of two randomized studies examining women with overweight or obesity and PCOS and the effect of lifestyle intervention before ovulation induction versus immediate ovulation induction on infertility (16). The authors found that preconception weight loss with lifestyle interventions significantly improved ovulation and live birth rates compared to immediate ovulation induction (62.0% vs. 44.7%, RR 1.4 (1.1–1.7), *P* = 0.003; 25.0% vs. 10.2%, RR 2.5 (1.3–4.7), *P* = 0.01, respectively) (16). Lifestyle intervention consisted of caloric restriction, meal replacements, anti-obesity medication, behavioral modification, and increased physical activity leading to approximately a 5.4 kg weight-loss from the intervention prior to receiving ovulation induction (16).

Current literature supports weight loss as a tool to improve fertility in women with obesity and PCOS. In a study of 24 women with PCOS and obesity or overweight, a low-calorie, low-fat diet was implemented for 6–7 months. Of the 13 women who lost > 5% of their initial weight, 11 had menstrual dysfunction in which 9 showed improvement in regards to menstrual function and 5 conceived. In women who lost < 5% of their weight, only 1 out of 8 women showed improvement in menstrual function (4).

The exact mechanism by which a KD improves fertility is unknown and large clinical trials evaluating this have yet to be performed. Possible mechanisms are hypothesized to be associated with improvements in reproductive hormones and insulin resistance which are known to play a key role in the pathogenesis of PCOS and impaired ovulation.

Insulin and other inflammatory factors play a negative role in enhancing the production of Androgens especially Testosterone by the ovary. Reducing insulin levels are well as other inflammatory factors would slow that enzymatic reaction and improve ovarian function (17).

A meta-analysis including 170 women with PCOS on a KD for 45 days or more demonstrated reduced luteinizing hormone/follicle stimulating hormone ratio, reduced serum free testosterone, and increased sex hormone binding globulin (18). In another meta-analysis that compared insulin resistance markers in women with recurrent pregnancy losses with healthy women, women with recurrent pregnancy losses had significantly higher fasting plasma insulin, higher HOMA-IR, and lower glucose to insulin ratio (19). Furthermore, in a small study in which women with PCOS who previously failed an in vitro fertilization cycle were placed on a KD, markers of insulin resistance and fertility rates were evaluated pre and post intervention. KD resulted in significant improvements in fasting insulin, HOMA-IR, implantation (83.3% vs. 8.3%) and clinical pregnancy (66.7% vs. 0%) (20).

Diets other than KD are reported to have benefits in patients with PCOs. Low-carbohydrate diet (carbohydrates accounts for less than 45% of the three major nutrients) demonstrated significant reductions in BMI, HOMA-IR, total cholesterol, LDL and T levels while increasing FSH and SHBG levels (21). The question of whether a specific type of diet improves fertility in women with PCOS more than others is unknown. In a randomized trail, Mediterranean/low carbohydrate diet (maximum carbohydrate intake of less than 20%, a maximum carbohydrate intake of 100 g throughout the day and an increased intake of protein and fat) showed similar metabolic benefits when compared to low fat diet (less than 30% of total dietary calories from fat, less than 40 g of fat intake throughout the day and up to 10% saturated fat). However, no significant different in restoration in regular menses between both diets (22).

Head-to-head studies comparing KD to other diets in patients with PCOs are scarce. In a recent study that randomized 27 patients with PCOs to mediterranean diet versus very low-calorie keto diet (VLCKD) for 16 weeks, reductions in BMI, waist circumference, fat mass, and blood pressure were significantly higher in the VLCKD arm. No differences in fasting insulin, HOMA-IR, total cholesterol, HDL, Triglycerides, AST or hirsutism between the two arms. However, VLCKD resulted in significant increase in sex hormone binding globulins (SHBG) and free testosterone. Psychological stress and well-being were similar between both groups. Ovulation improved significantly in the VLCKD group, however, changes in pregnancy rates were not examined (23).

In a similar study to ours, 17 women with PCOS underwent mixed ketogenic diet for 45 days. The study diet protocol allowed a maximum daily carbohydrate intake of 30 grams and daily lipid intake of 30 grams, but restricted daily calorie intake to 600 kcal. The study resulted in a dramatic average weight loss of 9.4%. Twelve participants had restoration of regular cycles with 5 of them achieving pregnancy. However, the correlation between the degree of weight loss and the restoration of regular menstruation and pregnancy was not explored. Furthermore, the role of metformin and ovulation induction use was not studied (24). In our study, women on a KD for at least 3 months achieved > 5% weight loss at the time regular menses resumed and at conception. The rate of improvement in menstruation (100%) and pregnancy (55.6%) is considerably higher in our study compared to prior studies even when metformin and ovulation induction were used concomitantly. Furthermore, there were 5 women who achieved pregnancy without the use of ovulation induction agents (only one was on metformin). Two of them lost < 5% of their body weight at the time of conception and two other women had BMIs > 40. Based on current literature, these factors generally impede ovulation and pregnancy. Thus, we hypothesize that a physiologic change besides weight loss that the KD induces, such as improvement in insulin resistance, is the key to improving fertility in this population.

The gold standard technique for measuring insulin resistance, euglycemic insulin clamp studies, have not been conducted in women with PCOS who are placed on a KD. Surrogate markers of insulin resistance such as HOMA-IR, fasting insulin, HDL, and triglycerides have been evaluated in small studies. Paoli et al. conducted a twelve week, single-arm, prospective study evaluating the metabolic effects of a KD on overweight women with PCOS. The study found that the mean weight reduction was 12%, HOMA-IR decreased significantly before and after the intervention (2.85 ± 0.15 vs. 2.32 ± 0.13; *P* < 0.0001) as well as serum triglycerides (2.31 ± 0.40 vs. 1.87 ± 0.27 mmol/L; *P* < 0.0008), HDL (1.79 ± 0.41 vs. 2.02 ± 0.43 mmol/L; *P* = 0.0146), and insulin (12.62 ± 0.48 vs. 11.31 ± 0.60 μU/mL; *P* < 0.0001) (8). Another study in which women with BMI ≥ 27 kg/m2 and PCOS were placed on a KD for 24 weeks, demonstrated significant reductions in weight (12.1%) and fasting insulin (−54%) while maintaining normal fasting glucose levels (7). Serum triglycerides and HDL did not significantly differ. In a recent study by Magaganini et al. (25), 25 patients with obesity and PCOS underwent VLCKD for 12 weeks. At the end of the study, 76% of patients switched from obesity to overweight, 96% of participants had normalization HOMA-IR, serum AMH levels significantly decreased, and progesterone and SHBG significantly increased after VLCKD. The rates of ovulation or pregnancy were not examined (25). All of these studies are small (*n* = 14, 5, and 25 respectively) and do not address fertility outcomes, but the KD has a profound effect on weight loss and improving metabolic parameters of insulin resistance.

Although euglycemic insulin clamp studies (clamp study) have not been performed in the PCOS population, a short-term, small, clamp study involving 10 patients with type 2 diabetes (T2DM) and obesity showed that insulin resistance improved on a KD. Patients had weight and metabolic parameters including serum insulin checked at the end of the 7-day control period (usual diet) and at the end of the 14-day KD period. Mean 24-hour serum insulin level was statistically significantly lower after the end of the KD period compared to the usual diet period demonstrating the insulin sensitizing effect of KD (26). Furthermore, an open-labeled, non-randomized, controlled study of 359 patients with T2DM who were started on a KD and followed for 1 year, demonstrated an earlier decrease in insulin resistance markers compared to weight loss. The significance of change during the first 70 days of the intervention and the next 295 days was compared. The percentage of effect that the first 70 days on the KD had on weight was 62% compared to 73% for serum insulin and 87% for HOMA-IR suggesting that a KD improves insulin resistance before weight loss becomes apparent (27). This earlier response in insulin resistance reduction may be the reason for improved fertility despite minimal weight loss seen in some of the patients in our study.

KD is reported to have positive impact on inflammation and comorbidities and health outcomes related to PCOs. Ketone bodies, specifically, β-hydroxybutyrate, are found to have anti-inflammatory effects through inhibiting interleukins and anti-oxidative effects (28). This anti-inflammatory role alongside lipids, body weight and blood pressure reduction are thought to contribute significantly to the KD role in cardiovascular protection (28). KD is shown to have a beneficial effect in patients with type 2 diabetes by reducing oral intake along with concordant reductions in insulin requirements and the amount of other anti-diabetes therapies (29). Beyond anthropometric, metabolic and endocrine parameters, KD have promising effects on health related quality of life, social behavior (30) and mental health disorders including schizophrenia and bipolar disorders (30, 31). Improvements in other health outcomes associated with PCOS may also have influenced the observed fertility.

Studies examining the long-term effects of a KD on fertility are lacking and its effects on metabolic/reproductive markers are scarce. In a meta-analysis comparing KDs with a low-fat diet in patients with obesity over a period of 12 months or more, participants assigned to a KD had statistically lower body weight [weighted mean difference −0.91 (95% IC −1.65, −0.17) kg], lower triglyceride levels [weighted mean difference −0.18 (95% CI −0.27, −0.08) mmol/l], lower diastolic blood pressure [weighted mean difference −1.43 (95% CI −2.49, −0.37) mmHg], higher LDL-C [weighted mean difference 0.12 (95% CI 0.04, 0.2) mmol/l], and higher HDL-C [weighted mean difference 0.09 (95% CI 0.06, 0.12) mmol/l] (32). Although statistical significance was shown for these parameters, the clinical significance of, for example, a weight reduction of 0.91 kg is questionable.

Because of the general lack of long-term studies on KD, its adverse effects of the KD over the long-term are not well known. Difficulty with adherence to a KD due to its strict dietary restrictions may play a role in this (33). In rodents, long-term maintenance KD precipitates the development of non-alcoholic fatty liver disease as well as glucose intolerance (34).

In a randomized trial of 100 women with PCOS but no obesity, the ovulation rate at 6 months was 100% in the metformin group and 37% in the placebo group (35). The number of menstrual cycles over a 6-month period per patient was 4.6 in the metformin group compared to 2.4 in the placebo group (P < 0.001) (35). Overall, past trials have been consistent with these findings where metformin monotherapy increases ovulation rate in women with PCOS (36).

In terms of pregnancy rates, existing studies in which a metformin group was compared to a placebo group were underpowered and did not provide meaningful results. However, a meta-analysis performed by Tang et al. suggested mild improvement in pregnancy rates in the metformin group compared to placebo (OR 2.31; 95% CI 1.52–3.51) (37).

Our study demonstrated return of regular menstrual cycles in all patients regardless of metformin use and resulted in 100% pregnancy rate in the non-metformin group compared to 41.7% in the metformin group (P = 0.036). Four out of the 5 patients in the non-metformin group were not using any ovulation induction agents as opposed to the metformin group in which 4 out of 5 patients were using an ovulation induction agent. The difference in pregnancy rate may be explained by the slightly heavier initial weight of the metformin group leading to potentially more severe insulin resistance and resistant anovulation. Furthermore, the ability to achieve regular menstruation and pregnancies without the aid of metformin can likely be attributed to weight loss and improvements in insulin resistance.

In our study, 7 of the 8 patients receiving ovulation induction agents who desired pregnancy were also on metformin, and 62.5% of them achieved pregnancy. Four patients used clomiphene, 5 used letrozole, and 1 used both clomiphene and letrozole. The one patient who was not on metformin used letrozole alone. Combination therapy of metformin and clomiphene citrate resulted in a higher rate of conception compared to metformin alone (38.3% vs 12.0%, P < 0.001) and to clomiphene citrate alone (38.3% vs 29.7%, P = 0.003) (11).

There is no data regarding the combination use of metformin and letrozole, but letrozole monotherapy has been shown to improve pregnancy rates more than clomiphene citrate monotherapy (31.3% vs 21.5%, P = 0.003) (12). Compared to previous studies using combination therapy, our pregnancy rate of 62.5% appears to demonstrate a higher rate of success. Again, weight loss and improved insulin resistance, in addition to pharmacotherapy, are the likely drivers to achieving a higher successful pregnancy rate.

In women, AMH is produced by the granulosa cells of the pre-antral and small antral follicles (38). Based on animal studies, AMH seems to inhibit both follicle recruitment and growth, thereby regulating the pool size of follicles (39). Because AMH is essentially secreted by follicles that are in the stage immediately after recruitment from the primordial pool and just before selection, it has become a biomarker for ovarian reserve (39). AMH level usually declines with age and becomes undetectable at menopause (40).

In PCOS, AMH levels are elevated compared to those without (41). This is likely due to the increased number of AMH-producing pre-antral and small antral follicles in PCOS ovaries as well as increased production of AMH by individual granulosa cells (42). In women with PCOS, lower AMH levels are associated with better response to ovulation induction. A meta-analysis reported that BMI is negatively correlated with AMH in patients with obesity who had or didn’t have PCOS (43).

Mahran et al. studied women with PCOS and BMI ≤ 35 kg/m2 (mean 28.8) and found that serum concentrations above 3.4 ng/mL markedly reduced ovulation rates from 97% to 48% and pregnancy rates from 46% to 19% with use of clomiphene citrate (44). In a similar study, El Halawaty et al. reported that an AMH value above 1.2 ng/mL predicted lower ovulation rates with clomiphene citrate with a sensitivity of 71% and specificity of 65.7% in women with PCOS and BMI > 30 kg/m2 (mean 36.7) (41).

In our small sample of women with recorded AMH values, AMH ranged widely both within the pregnant group (3.4 to 10.8 ng/ml) and the non-pregnant group (0.1 to 23.7 ng/mL). Contrary to previous literature by Mahran et al. and El Halawaty et al., all of the women in this sub-cohort who became pregnant had AMH values greater than 1.2 ng/ml and 3 out of 4 had AMH values greater than 3.4 ng/ml (lowest AMH value was 3.4 ng/mL). Three out of 4 women did not require fertility induction agents to become pregnant.

The small sample size prohibits statistical comparison, but the non-pregnant group had a higher overall BMI compared to the pregnant group but HbA1c did not differ between the groups. The two women who were unable to become pregnant despite AMH values < 3.4 ng/mL and the use of ovulation induction agents, had BMIs of 47.5 and 54.0. As higher BMI is associated with lower AMH values, it is prudent to consider the effect of severely elevated BMIs when interpreting AMH values in women with PCOS.

The small sample size and retrospective nature of the study design may limit interpretation of our results. A more diverse population of women including varieties of ethnicities, different severities of PCOS, and different metabolic comorbidities would improve the generalizability of our findings. Adherence to the KD was also difficult to assess given the retrospective nature of the study. However, this is one of the first studies to report the effect of a KD on fertility outcomes in women with obesity and PCOS and the results are encouraging. A large prospective trial of a diverse population of women with obesity and PCOS comparing a control group (KD followed by ovulation induction) versus immediate ovulation induction group, with ovulation and pregnancy as primary outcomes, would provide critical insight into the effects of a KD on fertility. Improved health associated with PCOS may indirectly lead to better fertility rates. Assessment of improved overall health associated with PCOS were not assessed in the present study, and the study didn’t assess the effect of Ketogenic Diets on clinical outcomes associated with PCOS. Further studies to evaluate the association between improved health and fertility rates are necessary, as are studies that assess other clinical outcomes related to PCOS. An additional limitation of this report is the potential for residual confounding, a limitation that is inherent to all retrospective analyses. Only data that was documented and available to the investigators could be recorded and included in the analyses. The duration and intensity of exercise performed by the subjects was not recorded. Additionally, patients may have participated in other activities (mind-body practices) and/or consumed supplements outside of the nutrition recommendations they were provided. At present, the KD should be considered as an option to improve fertility in women with PCOS and obesity. However, future studies are needed to build a standardized KD protocol for this population to maximize efficacy and safety.

## 5 Conclusion

The KD may improve ovulation and fertility rates in women with PCOS regardless of metformin or ovulation induction use. This may be, in part, due to the weight loss and improvement in insulin resistance which may occur after initiation of a KD. Further prospective studies evaluating the impact of a KD on fertility appear warranted, as well as mechanistic studies to further elucidate the mechanism by which a KD may improve fertility, with or without weight loss.

## Data Availability

The raw data supporting the conclusions of this article will be made available by the authors, without undue reservation.
